# Design and characterisation of intervertebral disc mimicking phantoms for photoacoustic imaging^☆^^☆☆^

**DOI:** 10.1016/j.pacs.2025.100783

**Published:** 2025-12-10

**Authors:** Roman Allais, Valentin Espinas, Antoine Capart, Anabela Da Silva, Olivier Boiron

**Affiliations:** a Aix-Marseille University, CNRS, Centrale Méditerranée, IRPHE, Marseille, France; b Aix Marseille Univ, CNRS, Centrale Med, Institut Fresnel, Marseille, France

**Keywords:** Photoacoustics, Intervertebral disc, Phantoms

## Abstract

Photoacoustics has gained momentum as a new medical imaging technique owing to its ability to benefit from good optical contrast and acoustic resolution. To ease transfer into clinical settings and validate the algorithms, calibrated tissue-mimicking materials (TMM) are required. This paper describes a complete photoacoustic characterisation of a line of titanium dioxide (TiO_2_) doped agarose hydrogels whose optical absorption (490–835 nm), reduced scattering (590–815 nm), isobaric heat capacity, mass density, speed of sound and acoustic attenuation were quantified for an agarose concentration up to 4% w/w and a TiO_2_ concentration ranging from 0.25 to 1 mg/mL. Empirical constitutive laws as a function of the concentrations of the components were derived, enabling the creation of TMM with tailored properties. Results showed that these phantoms are suitable candidates to mimic the photoacoustic properties of various soft tissues including intervertebral discs (IVD). Photoacoustic probings performed on an IVD-mimicking phantom and six healthy porcine discs demonstrated the ability of these TMM to accurately replicate healthy IVD properties; this could serve as a first step towards an application of photoacoustic imaging to quantifying disc degeneration.

## Highlights

• The design of soft tissue phantoms with a focus on intervertebral discs is presented.
• The photoacoustics characteristics of soft tissue phantoms are determined.
• Empirical laws allowing the photoacoustic properties to be varied are proposed.
• Photoacoustic tests with excised tissues are carried out to valid the concept.

## 1 Introduction

Low back pain is a significant clinical and societal problem that affects the majority of the population at some stage in their lives. Additionally, a recent analysis identified low back and neck pain as the leading cause of years lost to disability worldwide [1]. This condition not only impacts the health and quality of life of patients but also imposes considerable societal and financial burdens; therefore, preventing and managing the condition is of prime interest. Although all the mechanisms related to low back pain are not fully understood, it is acknowledged to be correlated with intervertebral disc (IVD) degeneration [2]. This degenerative process starts quite early in life but typically remains asymptomatic for years. Its main dynamic is the progressive desiccation of the medium and after a few decades, pain might appear leading to a diagnosis made using qualitative grading schemes derived from MRI. Hence, there is currently a clinical need for a more quantitative and reliable diagnosis.

Photoacoustic imaging (PA) is a promising candidate because it enables non-invasive, quantitative and spectroscopic probing of soft tissues. PA can be summed up as a three-step process: a nanosecond light pulse is emitted on the medium to probe, where the absorbed light induces a brief thermoelastic expansion followed by an acoustic wave [3]; hence, PA combines both optical contrast and acoustic resolution. Indeed, optical techniques benefit from a good contrast based on the chromophores spectra but struggle to image deep tissues with sufficient resolution because of the high scattering of visible to near-infrared light in biological tissues. Conversely, sound is less scattered than light in biological tissues which enables ultrasound imaging to image deeper regions but at the cost of a lower contrast. PA has been shown a growing interest for the past twenty years in clinical applications such as oximetry [4] or cancer detection [5]. Additionally, preliminary studies on porcine lumbar discs suggest its potential utility in retrieving biological markers of IVD degeneration, namely, water and collagen concentrations [6], [7].

Before considering the use of photoacoustic imaging to quantify disc degeneration, tissue-mimicking materials (TMM) are particularly helpful as a first validation step. TMM has already proven to be useful for multiple applications, including the study of light-tissues interactions, serving as calibration references for established medical imaging devices or as validation for emerging imaging technologies or numerical models [8], [9]. Extensive research has already been conducted on the manufacturing and characterisation of purely optical phantoms of soft tissues, usually embedding an absorber (ink [10], [11], [12], specific dyes [11], [13], [14] ...) and a scattering agent (titanium dioxide [14], [15], [16], aluminium oxyde [12], [17], Intralipid® [10], [11], [13] ...) in a background medium. However, since liquid phantoms are unable to mimic layered structures and heterogeneous media, phantoms with a solid matrix are often preferred [10], [12], [14], [16], [18].

Literature related to multiphysical TMM characterisation is less abundant but is a growing research topic as new medical applications progressively integrate multiphysical interactions. In 2015, an acoustic and thermal characterisation of gelatin phantoms was published with the aim of mimicking soft tissues for a magnetic resonance-guided focused ultrasound application [19]. The authors measured the density, speed of sound, acoustic attenuation, specific heat capacity and thermal diffusivity for different gelatins and found values comparable to human tissues. Two years later, an other acoustics and thermal characterisation was proposed by Menikou and Damianou [20] who described two agar-based gels whose acoustic speed, attenuation, thermal diffusivity, conductivity and specific heat were similar to brain and muscle tissues for a similar application. Opto-thermal characterisation of some TMM can also be found, notably for photothermal applications. Jaime, et al. presented two phantoms, one with an agar matrix, the other with a polyvinyl chloride-plastisol (PVC-P) matrix, whose optical and thermal properties are similar to muscles and fatty tissues [21]. A noteworthy result is the use of NaCl to adjust the thermal properties without significantly altering the optical properties.

These partial characterisations can provide some insights for the development of photoacoustic TMM whose optical, acoustic and thermal properties should ideally be controlled. The optical characterisation consists in determining the absorption coefficient ($\mu_{a}$ in cm^−1^) and reduced scattering coefficient ($\mu_{s}^{\prime }$ in cm^−1^). The thermal aspect of photoacoustics is embedded in the Grüneisen parameter (Eq. (1)) which is a function of the volumetric coefficient of thermal expansion (β in K^−1^), isobaric heat capacity ($c_{p}$ in J K^−1^ kg^−1^) and acoustic velocity ($c_{s}$ in m s^−1^):

(1) $$\Gamma =\frac{\beta \;c_{s}^{2}}{c_{p}}$$

Finally, the ultrasound is governed by a wave equation, involving the mass density of the medium (ρ in kg m^−3^), the acoustic velocity and attenuation (α in dB cm^−1^).

In the specific application of photoacoustic imaging, TMM, also known as phantoms, should have controlled, stable and reproducible optical and thermo-mechanical properties, as close as possible to the properties of the tissue of interest. It can be a challenge to find or create such a material since a suitable phantom from an optical perspective is not necessarily a good candidate for the acoustic properties and *vice versa*. Previous studies demonstrated partial characterisations of TMM for photoacoustics. Spirou, et al. presented TMM whose speed of sound, density and acoustic attenuation were within 15% of human soft tissues reported values but the optical coefficients were only measured at 1064 nm and the speed of sound could not be easily adjusted [15]. A more comprehensive photoacoustic characterisation on two different poly(vinyl alcohol) (PVA) gels fabrication methods were proposed to mimic breasts to validate photoacoustic mammography [22]. The proposed TMM reached physiological values of the mass density, speed of sound and acoustic attenuation but the optical properties were investigated only in the near infrared (800–1200 nm) and the coupling between optics and acoustics *via* the Grüneisen parameter was not explored. Conversely, Cabrelli, et al. managed to create TMM with physiological optical absorption in the 400–1200 nm window but optical scattering and mass density were significantly lower than expected values for most of the soft tissues [23]. Meanwhile, a team created breast phantoms with a PVC-P matrix, ${\mathrm{TiO}}_{2}$ and black plastic colorant [18]. Despite a more complex manufacturing protocol, the optical and acoustic properties of these phantoms can be adjusted by varying their components concentrations, making these last TMM among the most refined although the thermal properties were not investigated. Notably, Fonseca, et al. were among the first to provide a characterisation of Γ for PVCP phantoms although their values of 1.01 ± 0.05 are significantly higher than in tissues [24]. Finally, recent works investigated new non water-based materials such as gel wax [25] and silk [26] as TMM with optical and acoustic properties close to soft tissues. Some of the reported advantages of such materials are their mechanical robustness and stability over time but their manufacturing is not straightforward and the gel wax recipe is proprietary which can potentially lead to batch-to-batch variability. Silk phantoms seems a promising new material as it does not require additives to reach physiological optical and acoustic properties, which can be useful in studies involving biocompatibility. Consequently, the literature highlights how challenging it is to create TMM that accurately mimic the physiological properties of biological tissues, with well-defined and independently adjustable optical, acoustic, and thermal properties.

Building on these previous works, we seek in this paper to create and fully characterise multiphysics phantoms of intervertebral discs relevant to a future application of disc degeneration diagnosis using photoacoustic imaging. IVDs are singular organs: healthy discs, which are devoid of vascularisation and innervation, are primarily composed of water, from 66% in the outer annulus to 84% in the nucleus [27], [28]. The other two main components are type I and II collagen and, to a lesser extent, glycosaminoglycans. As stated above, the main effect of disc degeneration is the progressive reduction of the water content, hence increasing the relative collagen concentration. This distinctive high, but decaying with time, proportion of water led us to choose an hydrogel as the phantom matrix. Moreover, an independent fine-tuning of the different properties would ease the design of relevant phantoms at different grade of degeneration.

Purified agarose hydrogels, forming clear and nearly colourless gels at concentrations below 2% w/w, therefore appeared to be promising candidates for such a matter. Thus, optical scattering could be tuned separately from optical absorption using ${\mathrm{TiO}}_{2}$. Agarose gels also have thermal and acoustic properties close to cartilaginous tissues due to the similar high water content [20], [29], [30], [31]. By extrapolating the regression derived by Campagnoli, et al. [29] which gives the volumetric specific heat of agar gels as a function of temperature and assuming a mass density of 1000 kg m^−3^, the computed calorific capacity is 4 J g^−1^ K^−1^. This estimation is comparable to the reported value of 3.6 J g^−1^ K^−1^ for cartilages [30], considering the measurement uncertainties detailed in these studies. Besides, the acoustic speed reported in agar gels with silica dioxide varies from 1485 to 1529 m s^−1^ [20] falling within the experimental values reported for the IVD, ranging from 1408 to 1661 m s^−1^ [31]. Finally, agarose hydrogels are biocompatible and the topic of numerous studies concerning cartilage regeneration. Based on the findings of those studies, collagen [32] or chondroitine sulfat [33], [34] could easily be incorporated to create more realistic IVD phantoms if need be.

We present here the development of a biocompatible, affordable and easy-to-produce hydrogel designed to mimic the optical, thermal and acoustics properties of intervertebral discs and similar tissues. Our objectives were threefold: (i) to provide a quantitative characterisation of $\mu_{a}$ and $\mu_{s}^{\prime }$ in the visible range, which is the window frequently used in photoacoustic measurements, as well as the mass density (ρ), speed of sound ($c_{s}$), acoustic attenuation (α) and isobaric calorific capacity ($c_{p}$) for agarose-${\mathrm{TiO}}_{2}$ hydrogels; (ii) to determine the optimal ranges of agarose and ${\mathrm{TiO}}_{2}$ concentrations to best replicate an IVD for an application in photoacoustic imaging; (iii) to validate the ability to use photoacoustics to probe IVD and IVD-mimicking materials. First, some reference data taken from the literature and our own measurements made on porcine discs are introduced, then the design and photoacoustic characterisation of the phantoms are presented and finally a comparison between the PA signals acquired on porcine IVD and an IVD-mimicking phantom is made.

## 2 Materials and methods

### 2.1 Reference data

**Fig. 1:**
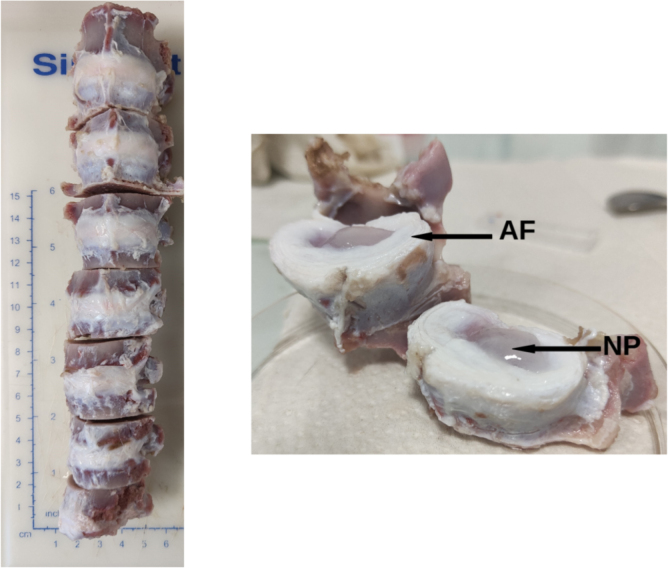
Left: the 7 intervertebral discs used for this study after the cleaning process; from top to bottom: L4/L5 - T11/T12. Right: example of IVD cut in the median transverse plane; *NP*: Nucleus Pulposus, *AF*: Annulus Fibrosus.

Intervertebral discs are heterogeneous media with two major compartments: the nucleus pulposus (NP), a viscous gel in the centre and the annulus fibrosus (AF) on the periphery, as illustrated on Fig. 1. Due to their differences in composition and structure, they are expected to have different optical and mechanical properties; easily tunable and versatile phantoms are therefore required to mimic these two compartments. However, the optical properties of intervertebral discs are scarce in the scientific literature. To remedy this situation, we expanded the validation data to cartilages, ligaments and synovialis which all share the same components in similar proportions [30] and have an histology comparable to IVD. This assumption was also made in an online database [35] referencing thermal, acoustic and electromagnetic properties of most of the human organs to help creating relevant numerical twins.

To complete our experimental dataset, we took as reference the speed of sound measured by Vayron and colleagues [31] and conducted our own measurements of the mass density and specific heat on annuli fibrosi. Briefly, the T11-L5 segment of the spine of an euthanised 6-month-old Duroc female swine was removed en-bloc by a qualified medical practitioner. All seven functional units were separated using an electric saw and the posterior process were cut. For each IVD, all the ligaments were carefully removed and the discs were abundantly rinsed with saline solution to remove residual blood (Fig. 1). Finally, each disc was put in an air-tight container filled with saline solution and stored in the freezer (≈−10 °C) until further use.

Prior to any measurements, the IVD were thawed at 4 °C during 24 h. To evaluate the density and specific heat, the annuli were carefully dissected and the excess of saline solution was wiped off. Each annulus was weighted using a standard laboratory precision-scale (± 0.1 mg). The volume of seven annuli was measured using a gas pycnometer (AccuPyc 1330, Micromeritics Instrument Corporation, Norcross, GA) and the isobaric heat capacity was computed on six annuli using a Dewar calorimeter (Jeulin, Evreux, France). The details of these two characterisations are presented in the next section.

### 2.2 Phantom design

**Fig. 2:**
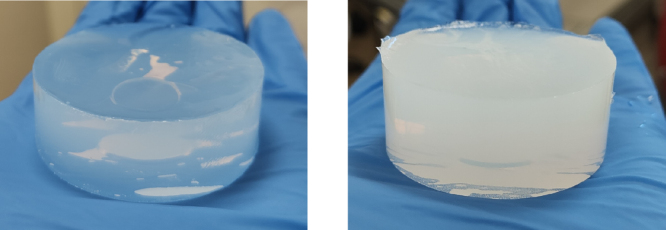
Left: representative phantom without ${\mathrm{TiO}}_{2}$. Right: representative phantom with ${\mathrm{TiO}}_{2}$.

For exploratory purposes, hydrogels with agarose concentrations $C_{agarose}\in \left[0.25,4\right]$ % (w/w) and with ${\mathrm{TiO}}_{2}$ concentrations $C_{{\mathrm{TiO}}_{2}}\in \left[0,1\right]$ mg/mL were made. This approach allowed to establish the empirical constitutive relationships for each parameter of interest as a function of the concentrations of the components, thus enabling the manufacture of phantoms with adjustable photoacoustic properties. For each hydrogel, the appropriate amount of agarose powder (Agarose (Low-EEO/Multi-Purpose/Molecular Biology Grade), Fisher BioReagents) and ${\mathrm{TiO}}_{2}$ powder (Titanium(IV) oxide, Sigma-Aldrich) were dissolved in 100 mL of distilled water and heated to 85 °C under magnetic stirring to ensure an homogeneous solution. The solution was then allowed to cool down to 60 °C before being poured in moulds. Extreme care was made to avoid the creation of bubbles during heating and pouring; notably, we used a syringe to transfer the phantoms into the moulds. Finally, the moulded hydrogels were left at room temperature (≈20 °C) during 30 min and then stored at 4 °C for gelation and conservation. Representative phantoms with and without ${\mathrm{TiO}}_{2}$ are shown on Fig. 2.

### 2.3 Phantom characterisation

All the above-mentioned phantoms were then characterised to find the concentrations that give the closest photoacoustic mimicry of an IVD. Prior to any of these characterisations, the phantoms were left at room temperature until the thermal equilibrium was reached, unless otherwise stated. Additional photographs and schematics of the experimental setups are provided in Supplementary materials.

#### 2.3.1 Optical absorption

We assumed ${\mathrm{TiO}}_{2}$ powder to act as a pure optical scatterer; hence, the absorption coefficient should solely be a function of $C_{Agarose}$. To measure the absorption coefficient of the agarose matrix at different concentrations (0.25%, 0.5%, 0.75%, 1%, 1.25%, 1.5% and 2% w/w), hydrogels without ${\mathrm{TiO}}_{2}$ were cast in standard spectrophotometer cuvettes (polystyrene, 10 mm pathlength) and characterised with a double-integrating-spheres setup (Ø50 mm Integrating Sphere, 3 Input Ports, M4 Tapped Mounting Hole, Thorlab, Newton, NJ). For each of the 7 hydrogels, the intensity of the reflected and transmitted light were measured at 15 different wavelengths with a photodiode amplifier (± 1 nA), from 490 nm to 835 nm with a 25 nm-step size and a 10 nm-bandwidth to get a spectrum for each concentration in the visible light. The light source was a NKTPhotonics SC400 white-light laser (NKT Photonics, Birkerød, Denmark) with a NKTPhotonics SuperK Varia monochromator (NKT Photonics, Birkerød, Denmark). The experimental setup and more details on the protocol can be found in the literature [36].

The post-processing was performed using the inverse-adding-doubling (IAD) algorithm developed by Prahl and coworkers [37] and their Python toolbox [38]. The algorithm requires the refractive indices of the medium and cuvette and the anisotropy factor (g) of the medium as inputs. The refractive index of the hydrogels and polystyrene were respectively set to 1.335 [12] and 1.59 and variations with concentrations and wavelengths were neglected. A sensitivity analysis showed that the outputs of the IAD algorithm were not significantly sensitive to the input value of g. Therefore, we arbitrarily set g=0.99 and considered the agarose gels to be quasi-non-scattering.

#### 2.3.2 Optical scattering

Unaccounted light losses in the IAD algorithm lead to unreliable results for the agarose-${\mathrm{TiO}}_{2}$ TMM characterisation, even for samples of relatively low thickness. Consequently, we used an other setup based on the integral reflectance model [39] to compute the reduced scattering of TMM with ${\mathrm{TiO}}_{2}$, with the light source varying from 590 nm to 815 nm with a 25 nm-step size and a 10 nm-bandwidth. Briefly, the experiment consisted in illuminating the samples in a quasi-normal incidence and taking a picture of the light spot using a 16-bit camera (Hamamatsu ORCA Flash 4.0, Hamamatsu Photonics, Hamamatsu City, Japan). Then the picture was post-processed using an in-house Python code to compute the integral reflectance and to fit the empirical model of Gobin, et al. [39]. This experiment works under the diffusion approximation and also allows to retrieve both the absorption and reduced scattering coefficient. As a preliminary validation, the absorption coefficients obtained were compared with the results of the integrating spheres and showed a good agreement. *In fine*, for this characterisation, the agarose-${\mathrm{TiO}}_{2}$-phantoms were cast in cylindrical moulds (diameter = 40 mm, height = 19 mm). Three agarose concentrations (0.75%, 1% and 1.5%) and five ${\mathrm{TiO}}_{2}$ concentrations (0.25 mg/mL, 0.35 mg/mL, 0.5 mg/mL, 0.65 and 1 mg/mL) were investigated, resulting in 15 different combinations. To assess the repeatability of the manufacturing and experiment, 4 combinations of ($C_{Agarose}$, $C_{{\mathrm{TiO}}_{2}}$) were also made in triplicate.

#### 2.3.3 Isobaric heat capacity

Isobaric heat capacity of the hydrogels and IVD was measured with a Dewar calorimeter (Jeulin, Evreux, France). This experiment relies on the first law of thermodynamics: a medium of known temperature ($T_{phantom}$) and mass but unknown calorific capacity is placed in water of known temperature ($T_{water}\neq T_{phantom}$) and mass inside the calorimeter. As a result, the two subsystems exchange heat until the thermal equilibrium is reached. A calibration experiment is also required to compute the experimental heat capacity of the calorimeter ($C_{calo}$). Finally, given the phantom mass ($m_{phantom}$), its temperature ($T_{phantom}$), the mass, temperature and isobaric heat capacity of water (respectively $m_{water}$, $T_{water}$ and $c_{water}$ (= 4.18 J K^−1^ g^−1^)) and the equilibrium temperature ($T_{eq}$), the isobaric heat capacity of the phantom can be computed as:

(2) $$c_{p}=\frac{C_{calo}\;\left(T_{water}-T_{eq}\right)+m_{water}\;c_{water}\;\left(T_{water}-T_{eq}\right)}{m_{phantom}\;\left(T_{eq}-T_{phantom}\right)}$$

We used distilled water at room temperature ($T_{water}$= 20.9 ± 0.4 °C) and the phantoms (or annuli for the IVD characterisation) were added immediately after being taken out the refrigerator ($T_{phantom}$= 2.5 ±2.1 °C). All the temperatures were monitored using the thermocouple provided with the calorimeter (accuracy ± 0.1 °C). For each measurement, given the precision on the temperatures and masses, an estimation of the Type B uncertainty on $c_{p}$ was computed using the propagation law of uncertainties. Furthermore, for each phantom, three measurements were made on three different days to assess the repeatability and average the noise measurements. *In fine*, 25 combinations of agarose and ${\mathrm{TiO}}_{2}$ concentrations were characterised: $C_{Agarose}\in \left\{0.5,0.75,1,1.5,2\right\}$ % w/w and $C_{{\mathrm{TiO}}_{2}}\in \left\{0.35,0.5,0.65,0.8,1\right\}$ mg/mL. The same characterisation was performed on the annuli of 6 IVD; given the higher relative measurement uncertainties, 5 measurements were made on 5 different days for each IVD.

#### 2.3.4 Density

The mass of each sample was measured using a standard laboratory precision scale (± 0.1 mg) while its volume was determined with a gas pycnometer (AccuPyc 1330, Micromeritics Instrument Corporation, Norcross, GA); each volume was computed 3 times and their average was kept. 21 combinations of agarose and ${\mathrm{TiO}}_{2}$ concentrations were investigated: $C_{Agarose}\in \left\{0.5,0.75,1,1.5,2,3,4\right\}$ % w/w and $C_{{\mathrm{TiO}}_{2}}\in \left\{0.35,0.5,0.65\right\}$ mg/mL. The same protocol was applied to measure the density of the annuli, and the whole procedure was replicated three times on each annulus to assess the repeatability of the experiment on the IVD.

#### 2.3.5 Speed of sound

The acoustic properties of the phantoms were measured using a broadband through-transmission substitution technique [9], [16]. TMM samples were immersed in a tank filled with distilled water between two coaxial transducers (V358-SU-50, Panametrics, Waltham, MA) which were connected to a pulser-receiver (Model 5073PR, Panametrics). The bottom transducer acted as the emitter and the top transducer as the receiver; the pulse repetition rate was set to 200 Hz, the energy to 8 μJ, the damping to 17 Ω, the receiver voltage gain to 39 dB and the integrated filters were disabled. The signals were digitised at 4 GSa/s on a 100 MHz-oscilloscope (InfiniiVision DSO-X-3017A, Agilent Technologies, CA), averaged over 256 acquisitions to improve the signal-to-noise ratio and stored on an external hard-drive. For each sample, a calibration acquisition with only water in the tank was conducted. Subsequently, the sample, whose height (h) was measured using a caliper (± 20 μm), was carefully introduced in the tank between the transducers and a second acquisition was recorded. Knowing the speed of sound in water ($c_{w}$) as a function of the temperature [40] and the time delay between the two acquisitions introduced by the sample (Δt), the speed of sound in the sample can be computed as [41]:

(3) $$c_{s}=\frac{c_{w}}{1+\frac{\Delta t}{h}\;c_{w}}$$

*In fine*, 3 different samples of phantoms with $C_{Agarose}\in \left\{0.75,1,1.25,1.5,2,3,4\right\}$ % w/w without ${\mathrm{TiO}}_{2}$ were characterised to assess the repeatability of the experiment. Previous works stated that ${\mathrm{TiO}}_{2}$ does not significantly alter the acoustic properties in PVCP phantoms [15]. Phantoms with 0.8 mg/mL and 1 mg/mL of different agarose concentrations were also characterised to assess if their conclusion still holds for agarose phantoms.

#### 2.3.6 Acoustic attenuation

The experimental setup used to compute the speed of sound also allows to measure the acoustic attenuation. Assuming a total transmission at the interfaces between water and the hydrogels, the acoustic attenuation (α) as a function of the frequency was computed between 15 and 40 MHz as [41], [42]:

(4) $$\alpha \left(f\right)=-\frac{20}{h}\;log\left(\frac{A_{phantom}\left(f\right)}{A_{water}\left(f\right)}\right)+\alpha_{w}\left(f\right)$$

where $A_{phantom}$ is the magnitude of the signal spectrum, $A_{water}$ the magnitude of the calibration signal spectrum and $\alpha_{w}$ the acoustic attenuation of water [43]. A power law $\alpha =a_{0}f^{n}$ was then fitted to the data for each phantom, where $a_{0}$ and n are the fitted parameters. Finally, to quantify the potential influence of ${\mathrm{TiO}}_{2}$ on α, TMM with $C_{{\mathrm{TiO}}_{2}}$ = 1 mg/mL, corresponding to the highest concentration used to tune the optical scattering, were also characterised.

**Fig. 3:**
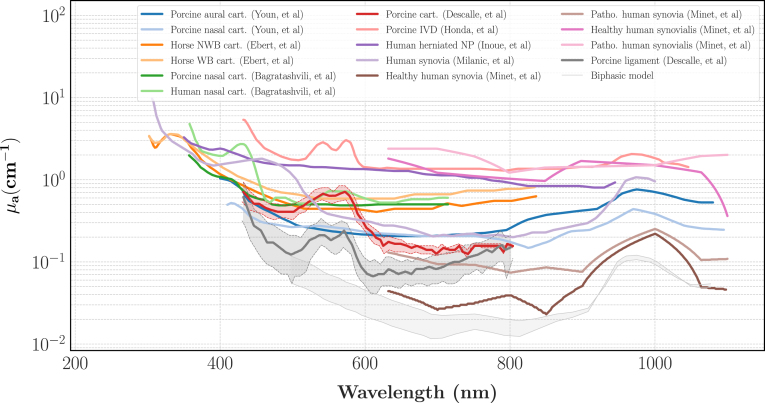
Literature review on the values of the absorption coefficient $\mu_{a}$ for various cartilaginous tissues. *Cart.*: cartilage, *NWB*: Non-Weight-Bearing, *WB*: Weight-Bearing. Youn, et al. [44], Ebert, et al. [45], Bagrastaschvili, et al. [46], Descalle, et al. [47], Honda, et al. [48], Inoue, et al. [49], Milanic, et al. [50], Minet, et al. [51]. The label *Biphasic Model* refers to an idealised model of an IVD solely made of water and collagen.

### 2.4 Photoacoustic probing

In order to assess the interest of the designed TMM for a future application in disc degeneration diagnosis, photoacoustic measurements were conducted on a phantom using the following experimental setup: a pulsed laser (NT352A, Ekspla, Lithuania, up to 120 mJ, scanning wavelength range 670–2600 nm + 532 nm, 20 Hz pulse repetition rate) served as the light source and the acoustic signal was acquired with an ultrasound transducer (Olympus, Japan, 1 inch diameter and 1 inch focal length), preamplified (Olympus, gain 40 dB, cutting frequency 10 MHz), digitised at 2.5 GSa/s on a 200MHz oscilloscope (Tektronix MDO3024, OR) and averaged over 512 acquisitions before being stored. The phantom was a cylinder (diameter = 40 mm, height = 19 mm) and a spectroscopic scanning was performed from 700 nm to 900 nm in 5 nm increments at the centre of the sample. Each signal was denoised using a Daubechies-wavelet denoising algorithm written in Python and normalised by the photodiode signal. The same spectroscopic scanning was performed on the anterior side of 6 IVD to compare the IVD-mimicking phantom with actual IVD.

## 3 Results

Unless otherwise stated, the results in this section are expressed as mean ± 1 standard deviation and the expanded uncertainties are computed with a coverage factor k = 1.96.

### 3.1 Reference data

#### 3.1.1 Optical coefficients

**Fig. 4:**
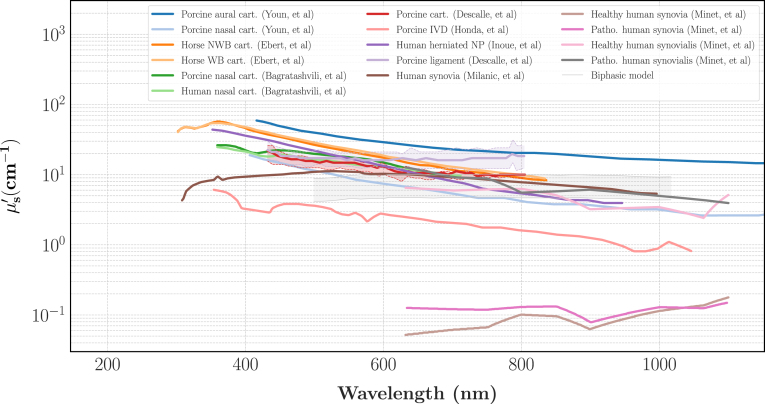
Literature review on the values of the reduced scattering coefficient $\mu_{s}^{\prime }$ for different cartilaginous tissues. The references are the same as in Fig. 3.

We reported on Fig. 3, Fig. 4 the optical spectra in the visible light of some cartilages, ligaments, synovialis and IVD previously characterised in the literature. The label *Biphasic Model* refers to an idealised model of an IVD solely made of water and collagen. For this model we assumed the annulus had a water volume fraction ($\Phi_{water}$) of 66% and the nucleus a water volume fraction of 84% [27], [28] and given the optical spectra of pure collagen and water [52], the spectra of this idealised IVD can be computed with a mixing law as:

(5) $$\mu_{a,biphasic}=\;\Phi_{water}\;\mu_{a,water}+\left(1-\Phi_{water}\right)\;\mu_{a,collagen}$$

(6) $$\mu_{s,biphasic}^{\prime }=\;\left(1-\Phi_{water}\right)\;\mu_{s,collagen}^{\prime }$$

As illustrated, there is a considerable variability in the literature concerning the absorption coefficient, with values spanning two orders of magnitude from 0.01 to 1 cm^−1^ at 700 nm, the lowest values given by the idealised model. In addition to the inherent biological variability, differences in experimental setups and post-processing algorithms used to do the characterisations are likely to affect the results. Moreover, it is well-known that tissue preparation can significantly alter the optical spectra. For instance, the cartilages and ligaments absorption spectra presented by Descalle, et al. [47] and the IVD absorption spectrum presented by Honda, et al. [48] exhibit the characteristic double-bell shape of the haemoglobin spectrum [53]. Thus, it is likely that the samples used is those studies suffered from blood staining which would yield an overestimation of $\mu_{a}$. Given these considerations, the spectra of Descalle et al. and Honda et al. were considered as outliers and removed from our analysis. For our purposes, we believe that an appropriate range. that an appropriate target range for healthy IVD would be $\mu_{a}\in$ [0.1, 0.6] cm^−1^ in the visible light.

Fig. 4 shows the reduced scattering spectra taken from the literature. Exception made for the synovia characterisation of Minet, et al. [51] which gives oddly low values of $\mu_{s}^{\prime }$, there is relatively less variability in the reduced scattering spectra. The idealised model also gives lower values than the experimental data, which can also be explained by staining and an oversimplified model. When averaging these spectra, [5, 20] cm^−1^ appeared as a suitable range for $\mu_{s}^{\prime }$ in the targeted range of wavelengths.

#### 3.1.2 Specific heat capacity

**Fig. 5:**
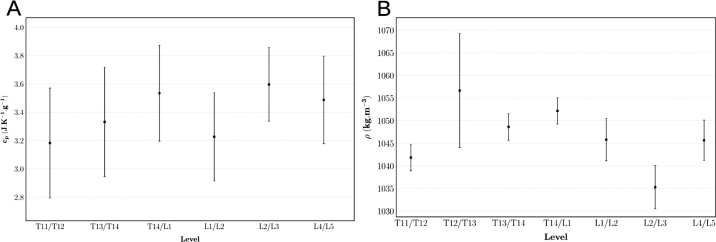
Experimental data of IVD specific heat (A) and density (B) along the spine. For each level, the average value over 3 measurements ± 1 expanded uncertainty is displayed.

Fig. 5.A shows the mean value of $c_{p}$ for each level; T12/T13 was excluded from the analysis due to insufficient tissue collection, resulting in unreliable measurements. For the remaining IVD, the average measurement uncertainty was 4.43% and the repeatability over 5 trials was good (mean CV<6.5%). Across the 6 IVD, we computed a specific heat of 3.40 ± 0.26 J K^−1^ g^−1^, which is also comparable to the 3.50–3.60 J K^−1^ g^−1^ reported for cartilages and the 3.36 J K^−1^ g^−1^ of ligaments and tendons [30].

#### 3.1.3 Density

Fig. 5.B shows the mean value of ρ measured at each intervertebral level. The average measurement uncertainty was 0.28% and the average coefficient of variation (CV) over the three measurements was inferior to 1%, indicating an excellent repeatability. We found that ρ varies from 1035 to 1056 kg m^−3^ with an average value of 1047 kg m^−3^, comparable to the mass density of 1100 kg m^−3^ reported for cartilages [30].

**Fig. 6:**
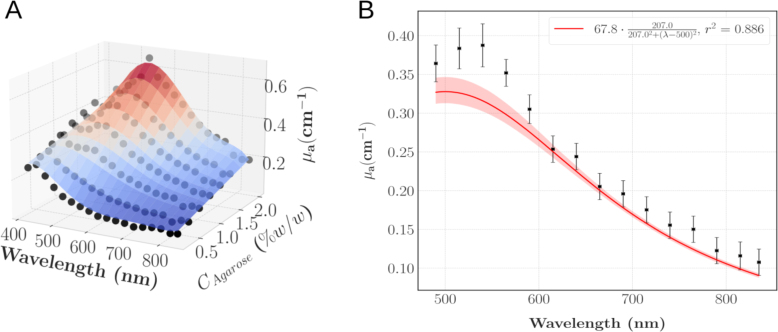
A: $\mu_{a}$ as a function of λ and $C_{Agarose}$. The black dots display the experimental datapoints; the spline interpolation is for illustrative purposes. B: Lorentzian function fit of $\mu_{a}$ as a function of λ for $C_{Agarose}$ = 1% w/w. The shaded area displays the 95% confidence interval.

**Fig. 7:**
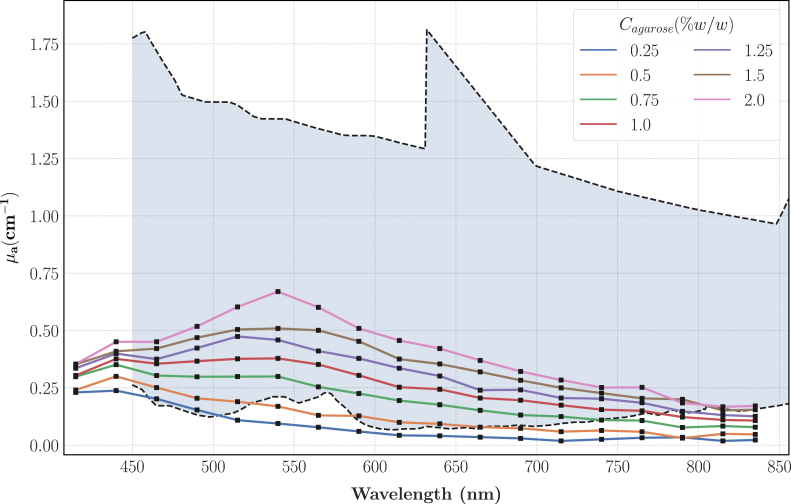
Experimental characterisation of $\mu_{a}$ for phantoms with different agarose concentrations and comparison with the results displayed on Fig. 3. The shaded area represents the envelope of Fig. 3 spectra; the spectra that showed clear blood staining were removed from the envelope computation. The black squares show the experimental points of the TMM characterisation and the measurement uncertainties are smaller than the squares.

### 3.2 Phantoms characterisation

#### 3.2.1 Optical absorption coefficient

Fig. 6.A displays the computed absorption coefficients for different agarose concentrations between 490 and 835 nm. As expected, the absorption linearly increases with the agarose concentration ($r^{2}=0.956\pm 0.028$) and because of the high water content in the phantoms, the optical absorption is in overall low. To derive the constitutive laws of $\mu_{a}$ as a function of λ and $C_{Agarose}$, a lorentzian function was fit to approximate $\mu_{a}$ as a function of λ. More precisely, for $C_{Agarose}\in$ [0.5, 2] % w/w and λ∈ [490, 835] nm, it can be approximated by:

(7) $$\mu_{a}\left(C_{Agarose},\lambda \right)=\left(a\;C_{Agarose}+b\right)\;\frac{c}{c^{2}+{\left(\lambda -500\right)}^{2}}$$

With the fitting parameters a, b and c as follow (mean ± standard error):

(8) $$\left\{\begin{array}{cc} \; & a=67.731\pm 0.426\;\text{nm cm}{}^{\mathrm{-1}}\text{(\%w/w)}{}^{\mathrm{-1}}\\ \; & b=0.099\pm 0.588\;\text{nm cm}{}^{\mathrm{-1}}\\ \; & c=207\pm 5\;\text{nm} \end{array}\right)$$

This model gives reliable predictions with an average coefficient of determination of 0.930 ± 0.067 and 0.863 ± 0.099 in respectively the ($\mu_{a}$, $C_{Agarose}$) and ($\mu_{a}$, λ) plans. Representative comparisons between the predicted values and the experimental datapoints are shown on Fig. 6.B. Fig. 7 compares the phantoms characterisation with the reference data. The shaded area represents the envelope of the spectra introduced as validation data for comparison on Fig. 3; the spectra flagged as outliers [47], [48], [51] were removed before the envelope was calculated since it would not have been representative of the true values of the tissues. Then, the envelope was defined by the minimum and maximum values of the remaining spectra at each wavelength.

Finally, propagation of the measurement uncertainties was estimated using a Monte-Carlo scheme: for each measurement 5000 trials of the IAD postprocessing were run. For each trial, all input values were sampled from a uniform law centered at the measured value and with a width of 10 nA, which corresponded to the resolution of our setup and taking into account the power fluctuations. Across the 126 measurements, the reliability was excellent (CV = 1.28% ± 1.24%).

#### 3.2.2 Reduced scattering coefficient

**Fig. 8:**
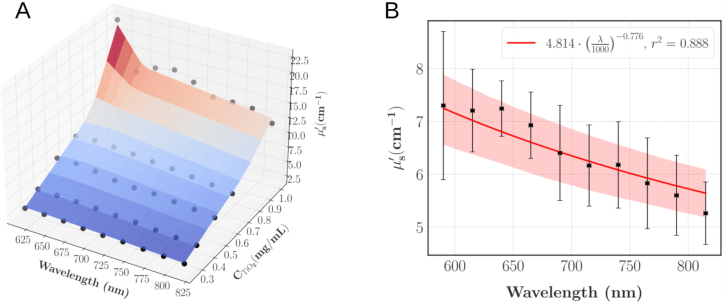
A: $\mu_{s}^{\prime }$ as a function of the wavelength (λ) and $C_{{\mathrm{TiO}}_{2}}$. The black dots display the experimental datapoints averaged over $C_{Agarose}$ (N = 7 samples for $C_{{\mathrm{TiO}}_{2}}\in \left\{0.35,0.65\right\}$, else N = 3); the spline interpolation is for illustrative purposes. B: Power law fit of $\mu_{s}^{\prime }$ as a function of λ for $C_{{\mathrm{TiO}}_{2}}$ = 0.5 mg/mL. The squares show the average values, the errorbars show ± 1 expanded uncertainty. The shaded area displays the 95% confidence interval.

The characterisation of the pure agarose hydrogels with the integrating spheres also revealed scattering to be negligible compared to the absorption. Consequently, we expected $\mu_{s}^{\prime }$ to solely depend on $C_{{\mathrm{TiO}}_{2}}$. This hypothesis was experimentally validated by our results: averaging over the agarose concentration yielded a coefficient of variation (CV = 10.03% ± 3.88%) below the repeatability threshold inherent to the model [39] and comparable to the 10% typical error reported for the regime $\mu_{s}^{\prime }\in$ [1, 15] cm^−1^. Hence, Fig. 8 depicts $\mu_{s}^{\prime }$ as a function of the wavelength and $C_{{\mathrm{TiO}}_{2}}$: $\mu_{s}^{\prime }$ was found to increase linearly with $C_{{\mathrm{TiO}}_{2}}$ for each wavelength ($r^{2}$ = 0.983 ± 0.009) and to decrease with λ. To quantify this last behaviour, a power law was fit as illustrated on Fig. 8.B which enables to compute $\mu_{s}^{\prime }$ for any wavelength of interest with good confidence. Given $C_{{\mathrm{TiO}}_{2}}\in$ [0.25, 1] mg/mL and λ∈ [590, 815] nm, the following optimisation problem was solved:

(9) $$\left\{\begin{array}{cc} \; & \underset{a_{C_{{\mathrm{TiO}}_{2}}},\;x_{C_{{\mathrm{TiO}}_{2}}},\;y_{C_{{\mathrm{TiO}}_{2}}}}{\min}(x_{C_{{\mathrm{TiO}}_{2}}}(a_{C_{{\mathrm{TiO}}_{2}}}\;{\left(\frac{\lambda }{1000}\right)}^{-4}\\ \; & \;+\;{\left(1-a_{C_{{\mathrm{TiO}}_{2}}}\right)\;{\left(\frac{\lambda }{1000}\right)}^{-y_{C_{{\mathrm{TiO}}_{2}}}})-\mu_{s}^{\prime }\left(C_{{\mathrm{TiO}}_{2}},\lambda \right))}^{2}\\ \; & a_{C_{{\mathrm{TiO}}_{2}}},\;x_{C_{{\mathrm{TiO}}_{2}},\;0},y_{C_{{\mathrm{TiO}}_{2}},\;0}=\left(0.5,\;5,\;2\right)\\ \; & \lambda \in \left[590,\;815\right]\;\mathrm{nm} \end{array}\right)$$

The parameter $a_{C_{{\mathrm{TiO}}_{2}}}$ quantifies the Rayleigh-to-Mie scattering ratio and systematically converged to 0 in our fits. Therefore the power law can be written as:

(10) $$\mu_{s}^{\prime }\left(C_{{\mathrm{TiO}}_{2}},\lambda \right)=C_{{\mathrm{TiO}}_{2}}\;x\;{\left(\frac{\lambda }{1000\;\mathrm{nm}}\right)}^{-y}$$

With the fitting parameters x and y (mean ± standard error):

(11) $$\left\{\begin{array}{cc} \; & x=9.629\pm 0.365\;\text{cm}{}^{\mathrm{-1}}\text{mL mg}{}^{\mathrm{-1}}\\ \; & y=0.775\pm 0.057 \end{array}\right)$$

On average, the relative error between the predicted values and the experimental datapoints was 6.92% ± 6.86% which is comparable with the accuracy of the integral reflectance model, hence validating our predictive model. From this fit, it can be deduced that the Mie regime is predominant (Rayleigh-to-Mie scattering ratio a = 0), therefore, as the final step of the optical characterisation, the anisotropy scattering coefficient (g) can be approximated using the Mie scattering theory given the size of the particles and refractive indexes. The refractive index of ${\mathrm{TiO}}_{2}$ as a function of the wavelength λ was computed as RefractiveIndex.info:

(12) $$n_{{\mathrm{TiO}}_{2}}^{2}=5.913+\frac{0.2441}{\lambda^{2}-0.0803}$$

Then, an open-source Python code (Scott Prahl’s Python code for Mie Scattering) was used to estimate the values of g between 590 and 830 nm for ${\mathrm{TiO}}_{2}$ particles. Computations were performed for particles with a radius of 125 to 250 nm with a step of 10 nm, wavelengths between 590 and 830 nm with a step of 1 nm and assuming $n_{Agarose}$ = 1.335. Since the size distribution of the ${\mathrm{TiO}}_{2}$ particles used in this study was not known, we assumed it followed the same distribution as in prevous studies [14]. On average, g showed little variations between 590 and 830 nm (g = 0.516 ± 0.045).

**Fig. 9:**
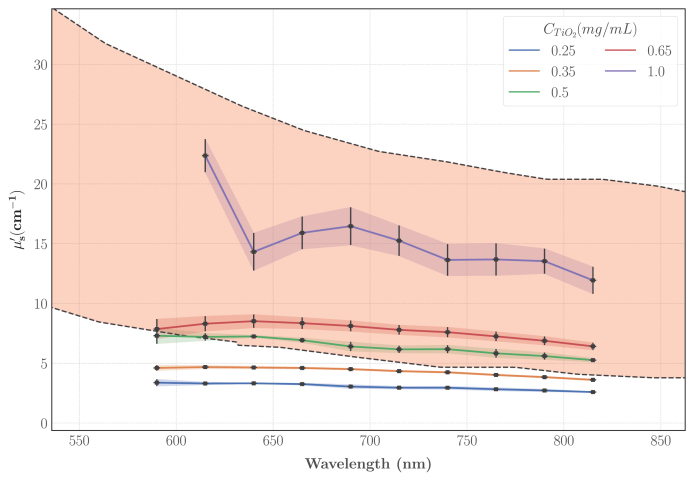
Experimental characterisation of $\mu_{s}^{\prime }$ for phantoms with different ${\mathrm{TiO}}_{2}$ concentrations and comparison with the results displayed on Fig. 4. The shaded area represents the envelope of Fig. 4 spectra; the spectra that showed clear blood staining and the synovia spectra were removed from the envelope computation. The black squares show the experimental points of the TMM characterisation; each datapoint is the average over the agarose concentrations and the errorbars show ± 1 standard error.

To compare this characterisation to the reference data specific to the IVD, the envelope of the spectra presented on Fig. 3 was computed after removing the data with clear blood staining and the synovia spectra which were considered as outliers. As illustrated on Fig. 9, the range of investigated ${\mathrm{TiO}}_{2}$ concentrations efficiently covers the lower half of the target values and given the quality of the fits, higher values of $\mu_{s}^{\prime }$ can be achieved by extrapolating the linear regressions. The calibration experiment at 590 nm for the phantoms with $C_{{\mathrm{TiO}}_{2}}$ = 1 mg/mL failed, explaining why there is a missing datapoint on Fig. 9.

Finally, the repeatability of the manufacturing and optical characterisation was quantified on 4 phantoms made in triplicate: the whole process demonstrated high repeatability (CV = 1.99% ± 1.57%), which is comparable to the 3% reported by Moffitt, et al. [14] who used integrating spheres and the inverse-adding-doubling method to compute the optical coefficients of a polyurethane phantom doped with ${\mathrm{TiO}}_{2}$.

#### 3.2.3 Specific heat capacity

**Fig. 10:**
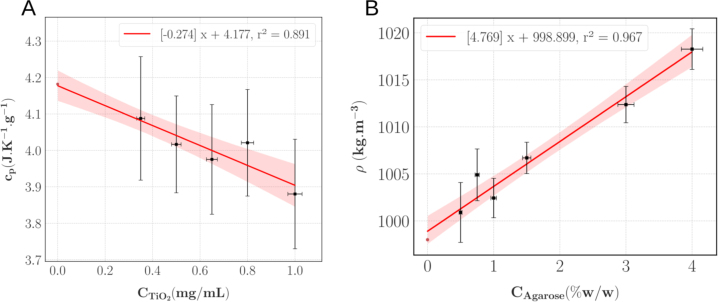
A: Specific heat as a function of $C_{{\mathrm{TiO}}_{2}}$. The squares show the average value over 15 phantoms (5 agarose concentrations × 3 measurements); the errorbars show ± 1 expanded uncertainty and the shaded red area the 95% confidence interval. B: Mass density as a function of $C_{Agarose}$. The squares show the average value over 3 phantoms (3 different ${\mathrm{TiO}}_{2}$ concentrations); the errorbars show ± 1 expanded uncertainty and the shaded red area the 95% confidence interval.

Our results revealed that the variability induced by the agarose concentration was too small to emerge from the measurement noise. Consequently, we considered $c_{p}$ to be independent from $C_{Agarose}$ in the range [0, 2] % w/w. However, we found $c_{p}$ to linearly decrease with increasing ${\mathrm{TiO}}_{2}$ concentration ($r^{2}$ = 0.891) as depicted on Fig. 10.A. Overall, the experiments had an average Type B uncertainty of 2.61% ± 0.81% and were highly repeatable over the three measurements (CV = 2.99% ± 1.74%). This repeatability on the measurement of the specific heat is comparable to previous characterisations on gelatin phantoms [19] made through the measurement of their thermal diffusivity and conductivity and on PVCP and agar phantoms whose specific heat was measured with a Differential Scanning Calorimeter (DSC), a more sophisticated experimental setup [21].

#### 3.2.4 Mass density

Due to the small amount of ${\mathrm{TiO}}_{2}$ powder in the phantoms, its impact on the mass density was insignificant; thus ρ is only function of the amount of agarose and can be averaged over the three ${\mathrm{TiO}}_{2}$ concentrations (CV = 0.20% ± 0.05%). The constitutive law of ρ as a function of $C_{agarose}$ is reported on Fig. 10.B: the mass density linearly increases with $C_{Agarose}$ ($r^{2}$ = 0.967).

#### 3.2.5 Speed of sound

**Fig. 11:**
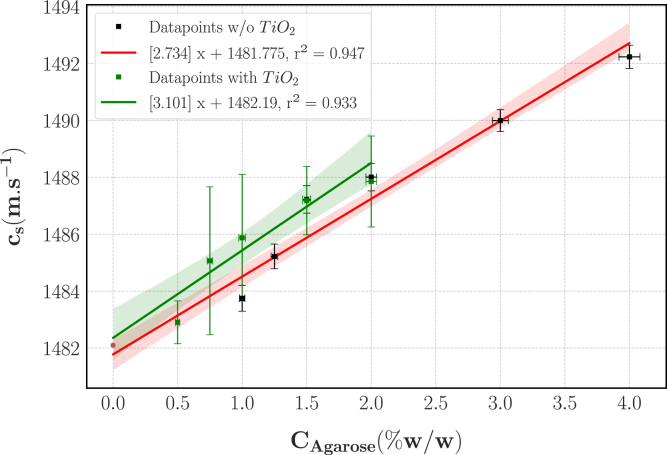
Speed of sound as a function of $C_{Agarose}$ with and without ${\mathrm{TiO}}_{2}$. The data are corrected to T = 19.9 °C. The squares show the average value over the three samples; the errorbars display ± 1 expanded uncertainty and the shaded areas the 95% confidence interval.

The speed of sound as a function of $C_{Agarose}$ with and without ${\mathrm{TiO}}_{2}$ is displayed on Fig. 11. As this characterisation was performed on three different days, the data of the last two days were corrected to account for the temperature dependency of $c_{w}$ and consequently $c_{s}$. Across the three days, the temperature varied from 19.8 to 21.3 °C; for each measurement, the temperature of the water was recorded (± 0.1 °C) and the associated $c_{w}$ was taken from the literature [40] to compute the speed of sound in the TMM according to Eq. (3). Finally, in order to correct the data from the experiments on day 2 and 3 to the temperature on day 1 (19.9 °C) we used the characterisation of Browne, et al. [54] who determined that the speed of sound in an agar TMM increases with temperature by 0.6 m s^−1^ °C^−1^ between 20 and 30 °C using Eq. (13) with $c_{s,\;raw}$ the computed speed of sound before the temperature correction and ΔT the difference of temperature between the day of the measurement and the reference temperature of day 1 (19.9 °C):

(13) $$c_{s}=c_{s,\;raw}-0.6\;\Delta T$$

Taking into account the measurements of the time of flights, TMM height and temperature, the average uncertainty on the speed was less than 1% (0.32 ± 0.14 m s^−1^). The speed of sound also exhibits a linear relationship with $C_{Agarose}$ ($r^{2}$ = 0.947) and these experiments showed an excellent repeatability (CV = 0.05% ± 0.00%). The results of the 0.5% agarose hydrogels without ${\mathrm{TiO}}_{2}$ showed aberrant values ($c_{s}\;<\;c_{w}$) and were considered outliers and thus discarded. This discrepancy likely comes from errors on the height measurement of the phantoms because of their extreme softness.

Finally, from the ρ and $c_{s}$ characterisations, the acoustic impedance $Z=\rho \;c_{s}$ can be computed. The computed $Z_{TMM}$ varies from 1.48 to 1.52 MRayl depending on the agarose concentration. As a result, the transmission coefficient between the phantoms and water $T_{w,TMM}$ varied from 0.987 to 1, validating our hypothesis of neglecting the acoustic losses at the interfaces for the computation of α.

#### 3.2.6 Acoustic attenuation

**Fig. 12:**
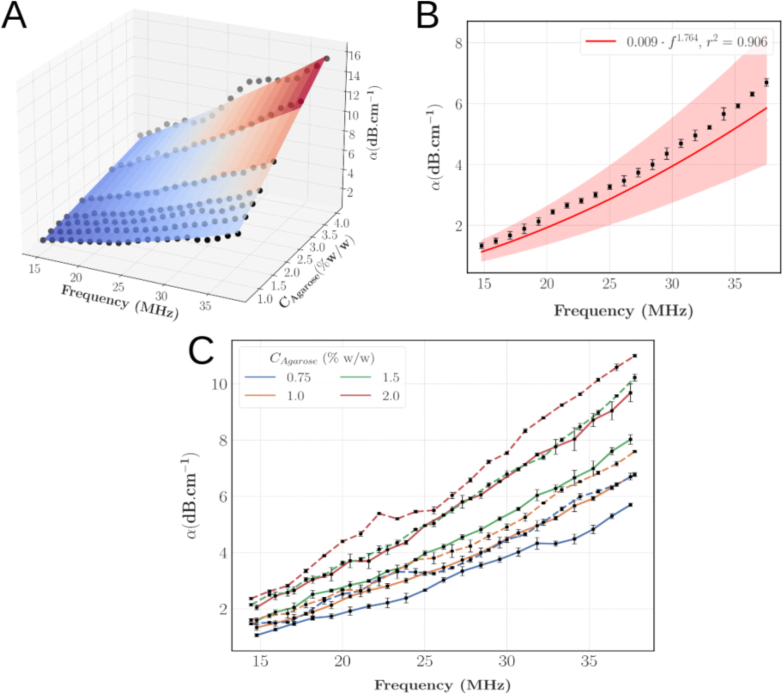
A: α as a function of f and $C_{Agarose}$. The black dots display the experimental datapoints; the spline interpolation is for illustrative purposes. B: Acoustic attenuation as a function of *f* for $C_{Agarose}$ = 1% w/w. The errorbars show ± 1 standard deviation. The shaded area displays the 95% confidence interval. C: Acoustic attenuation as a function of the frequency without (plain line) and with ${\mathrm{TiO}}_{2}$ (1 mg/ml - dashed lines) for different agarose concentrations. The squares show the mean values and the errorbars display ± 1 standard deviation.

The ultrasound signals induced by photoacoustic probing can have frequencies up to dozens of MHz, therefore a broadband characterisation of the acoustic properties is necessary when designing photoacoustic TMM. Overall, this characterisation also demonstrated good repeatability (average CV = 3.37% ± 2.58%). The acoustic attenuation between 15 and 35 MHz for phantoms of different agarose concentrations is shown on Fig. 12.A. Qualitatively, α increases with the frequency and $C_{Agarose}$. For each agarose concentration, a power law best fit ($r^{2}$ = 0.994 ± 0.008) was computed on the average spectrum (over three phantoms) between 15 and 35 MHz, which subsequently enabled to retrieve the constitutive relationship of α as a function of the frequency f in MHz and $C_{Agarose}\in$ [0.75,4] % w/w:

(14) $$\alpha \left(C_{Agarose},f\right)=\left(a_{2}\;C_{Agarose}^{a_{3}}+a_{1}\right)\;f^{b_{2}\;C_{Agarose}+b_{1}}$$

With the fitting parameters $a_{1}$, $a_{2}$, $a_{3}$, $b_{1}$ and $b_{2}$ (mean ± standard error):

(15) $$\left\{\begin{array}{cc} \; & a_{1}=0.0072\pm \;0.0010\;\text{dB cm}{}^{\mathrm{-1}}\text{MHz}{}^{-b}\\ \; & a_{2}=0.0026\pm 0.0005\;\text{dB cm}{}^{\mathrm{-1}}\text{MHz}{}^{-b}\text{(\% w/w)}{}^{-a_{3}}\\ \; & a_{3}=2.636\pm 0.145\\ \; & b_{1}=1.897\pm 0.039\\ \; & b_{2}=-0.132\pm 0.019\;\text{(\% w/w)}{}^{\mathrm{-1}} \end{array}\right)$$

The model gives good predictions of the acoustic attenuation with an average coefficient of determination of 0.932 ± 0.045 and 0.951 ±0.014 in respectively the (α, $C_{Agarose}$) and (α, f) plans. Representative examples are shown on Fig. 12.B. Additionally, to investigate the effect of ${\mathrm{TiO}}_{2}$, phantoms with $C_{{\mathrm{TiO}}_{2}}$ = 1 mg/mL were characterised as well. As illustrated on Fig. 12.C, ${\mathrm{TiO}}_{2}$ increases the acoustic attenuation by 20.06% ± 8.52% across all frequencies.

#### 3.2.7 Grüneisen parameter

A good estimation of Γ is necessary for PA applications as it is used either as prior information in inverse problem resolution algorithms or as ground truth for reconstruction validation. The last required parameter for the computation of Γ is the volumetric coefficient of thermal expansion (β) which is complex to experimentally characterise and whose values are, consequently, scarce in literature for both soft tissues and TMM. First, estimations of the Grüneisen parameter for the nucleus pulposus ($\Gamma_{NP}$) can be computed using the speed of sound measured by Vayron, et al. [31] and assuming $\beta_{NP}\approx \beta_{water}$. Since $c_{s,NP}$ was measured at 25 °C [31], we take $\beta_{water}$(T=25 °C) = 0.258.10^−3^ K^−1^ [55] for consistency. As for the annulus fibrosus, $\Gamma_{AF}$ can be estimated using our measurements of ρ, $c_{s}\in$ [1489, 1652] m s^−1^, whose values were computed from the experimental measurements of the acoustic impedance [56], and $\beta_{AF}$ = 0.20–0.38.10^−3^ K^−1^ [57], corresponding to the values reported for bones and soft tissues. Finally, for the TMM, we will use the values of β measured on gelatin hydrogels [55]: $\beta_{TMM}$(T=20 °C) = 0.207–0.231.10^−3^ K^−1^. The computed values of Γ are reported in Table 1.

**Table 1:** Estimated values for Γ.

	Temperature (°C)	$c_{p}$ (J K^−1^ g^−1^)	$c_{s}$ (m s^−1^)	β (K^−1^)	Γ (-)
NP	25	4–4.1	1408–1661	0.258.$10^{-3}$	0.125–0.178
AF	≈ 20	3.13–3.65	1489–1652	0.20–0.38.10^−3^	0.121–0.331
TMM	≈ 20	3.87–4.1	1482–1493	0.207–0.231.10^−3^	0.111–0.133
TMM	≈ 37	–	–	–	0.178–0.213

#### 3.2.8 Photoacoustic measurements

**Fig. 13:**
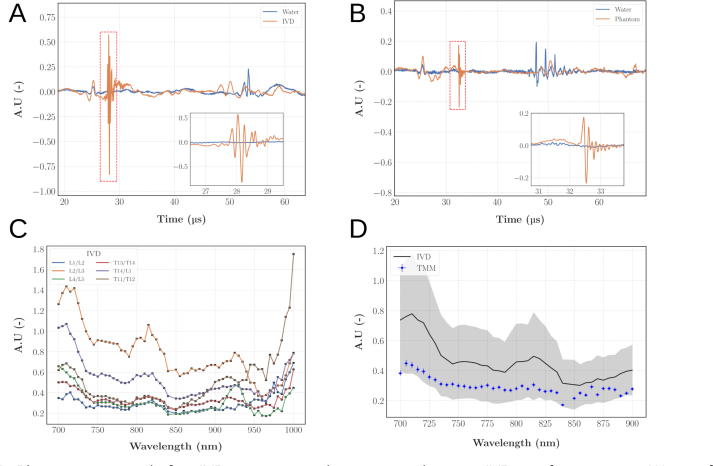
A: Photoacoustic signal of an IVD at 715 nm with a zoom on the water-IVD interface response; *Water* refers to an acquisition with only water in the tank. B: Photoacoustic signal of the phantom at 715 nm with a zoom on the water-TMM interface response. C: Peak-to-peak normalised amplitude spectra of the IVD. D: Mean ± 1 standard deviation of the IVD peak-to-peak spectra compared with the peak-to-peak amplitude spectrum of the TMM.

The photoacoustic measurements were conducted at room temperature (≈ 21.5 °C ± 0.5 °C) for both the IVD and TMM. Based on the results of the previously presented characterisation, we selected a TMM with $C_{Agarose}$ = 1.25% w/w and $C_{TiO2}$ = 1 mg/mL as a proxy to mimic an healthy IVD. Figs. 13A&B display representative signals obtained for respectively an IVD and the TMM for an illumination at 715 nm. The peak-to-peak (P2P) amplitude of the signal was computed and chosen as the validation metric: the P2P spectra of the IVD are shown on Fig. 13.C. Across the 6 samples, there is a moderate variability of the magnitude of the spectra which can be attributed to intrinsic biological disparity and some extent of blood staining. The pattern of the P2P spectra is also consistent with the optical absorption spectra of a collagen-water medium: the magnitude decreases from 700 to 900 nm and then starts to increase. Finally, as depicted on Fig. 13.D, the P2P spectrum of our TMM is comparable to the average spectrum of the IVD.

## 4 Discussion

We have designed and fully characterised a phantom mimicking the photoacoustic properties of an healthy IVD. To validate these properties, we relied on a thorough literature review of soft tissues of similar composition for the optical properties and the speed of sound, and we conducted our own measurements of the specific heat and density on porcine annuli to complete our dataset. We believe that taking properties measured on swines discs as reference data to be reliable, since porcine discs are similar to human discs in many aspects. Both have close compositions in terms of water content (67%–83% for humans [27], 71%–85% for pigs [58]), proteoglycans content (5–11 μg of uronic acid by mg of wet tissue for humans [27], 1–10 μg for pigs [59]) and collagen content (15%–58% of dry weight in human discs [60] *versus* 20.8–48.6 in porcine discs [58]).

### 4.1 Optical properties

The large deviation reported for the reference values (shaded areas on Fig. 6) can be imputed to several factors such as the inherent variability between biological samples or the preparation of the samples before their characterisation. The most critical contaminant is blood: due to its high absorption (≈ 200 cm^−1^ at 600 nm [53]), even relatively small amount of residual blood can alter the optical properties of a sample. Despite the overall low optical absorption of our TMM, the values span the lower half of the reference data and considering the quality of the fits, we are confident in extrapolating the regressions for agarose concentrations above 2% to achieve higher absorption. It is also interesting to note that the phantoms described in this paper are versatile enough to mimic soft tissues different from IVD and cartilages. The optical absorption is also analogous to the spectra of several other organs such as skin, prostate, breasts or bowels: the lowest agarose concentrations are able to reproduce the low absorption values of breasts or bowels in the near infrared, while higher concentrations can approach the values reached by skin and prostate [61].

The reduced scattering coefficient of our line of TMM is in the range of the reference data and showed the same behaviour as in previous studies: Moffitt, et al. [14], had a similar exponent (−0.8) of their power law fit of $\mu_{s}^{\prime }$ as a function of λ in their ${\mathrm{TiO}}_{2}$-polyurethane phantoms and they also reported a linear relationship with $C_{{\mathrm{TiO}}_{2}}$. However, even if both agarose and polyurethane were considered as non-scattering, there is significant mismatches between the linear regressions slopes: we obtained slopes 81% and 57% higher at respectively 690 and 830 nm. Such differences are likely explained by the higher refractive index of polyurethane: $n_{Polyurethane}$ = 1.468 against $n_{Agarose}$ = 1.335. As a result, it decreases the difference in refractive indexes between the background matrix and ${\mathrm{TiO}}_{2}$ particles and consequently the reduced scattering coefficient of the phantom. We completed the optical characterisation with a theoretical computation of g using Mie theory. It appeared that our approximation of the anisotropy coefficient falls short when compared to soft tissues which are usually in the range 0.7–0.99 [53] but our TMM still match the reduced scattering coefficient of most soft tissues such as breasts, skin, prostate or bowels [61].

Although the present optical characterisation covers the range 400 to 835 nm, higher wavelengths such as 1064 nm are commonly used in photoacoustics applications. At this wavelength, the main absorber is water with an absorption coefficient of $\approx 0.10\;{\mathrm{cm}}^{-1}$. Therefore, we expect $\mu_{a}$ to remain relatively stable between 835 and 1064 nm with a slight increase for the phantoms with agarose concentrations below 1% w/w. The reduced scattering coefficient can be extrapolated using Eq. (10) assuming the Mie regime still applies.

Finally, the ability to tune $\mu_{a}$ and $\mu_{s}^{\prime }$ independently within the reported ranges for physiological tissues and their relatively low baseline values make our phantoms as promising candidates for the design of future studies, as dyes could be incorporated during the manufacturing to reach any desired values.

### 4.2 Thermo-mechanical properties

Despite the high water content of the hydrogels, the addition of ${\mathrm{TiO}}_{2}$ brings the specific heat capacity close to some physiological values. As illustrated in Table 2 our hydrogels can mimic the specific heat of tissues such as liver, brain, prostate or cartilages with a relative difference of less than 10%. When comparing to the IVD, which is our primary target, our phantoms can accurately mimic the NP but the specific heat capacity we measured on the AF is still 14% lower. To get low enough values of $c_{p}$, an extrapolation of the regression presented on Fig. 10 gives a ${\mathrm{TiO}}_{2}$ concentration of 2.5 mg/mL. However this would lead to too high values of $\mu_{s}^{\prime }$: with this approach, a trade-off between $c_{p}$ and $\mu_{s}^{\prime }$ would be required. To avoid this trade-off, the thermal properties of the TMM could be more finely tuned by slightly changing the manufacturing protocol. While the use of graphite powder proved to significantly decrease the thermal conductivity and specific heat [62], its use in photoacoustic TMM is limited due to the alteration of the optical properties. Adding NaCl seems to be a more promising alternative as there is some evidence it can decrease the specific heat without significantly altering the optical properties [21].

The mass density of our TMM is comparable to the density of most of the soft tissues presented in Table 2 with relative differences typically less than 5% excepting for cartilages, skin and tendons. Concerning the comparison with the IVD, the difference of 30 kg m^−3^ (<3%) with our measurements on porcine AF should be negligible for most of the applications. Regarding the NP, we estimated its mass density to range from 923 to 1238 kg m^−3^ based on the measurements of the speed of sound [31] and acoustic impedance [56] taken from literature. Nonetheless, from our observations on the porcine discs, the NP are heavier than water, therefore mass densities of less than 1000 kg m^−3^ seem less likely.

**Table 2:** Comparison of some mechanical properties of biological tissues and our TMM.

	$c_{p}$ (J K^−1^ g^−1^)	ρ (kg m^−3^)	$c_{s}$ (m s^−1^)	Z (MRayl)	α (dB cm^−1^)
Water	4.18	998	1482	1.48	0.85 (20 MHz)
Brain	3.45–3.75 [30]	1030–1041 [57]	1525–1573 [57]	1.57–1.64	0.2–4.5 (1–5 MHz) [57]
Liver	3.6 [57]	1050–1070 [57]	1525–1639 [57]	1.60–1.75	0.5–0.7 (1–10 MHz) [63]
Breast	2.96 [30]	990–1060 [57]	1430–1570 [57]	1.42–1.66	0.5–12.6 (1.76–7 MHz) [57]
Prostate	3.74–3.78 [35]	1045 [57]	1614 [63]	1.69	0.2 (1–10 MHz) [63]
Tendon	3.36 [30]	1110–1220 [57]	1631 ± 36	1.77–2.03	3.7–19.3 (1–5 MHz) [57]
Skin	3.15–3.71 [57]	1093–1190 [57]	1503–1630 [57]	1.64–1.94	2.3–11.4 (1–5 MHz) [57]
Cartilage	3.5–3.6 [30]	1100 [30]	1627–1650 [35]	1.79–1.82	5–19 (1–5 MHz) [57]
A.F.	3.39 ± 0.26	1047 ± 10	≈ 1489–1652^a^	1.557–1.710 [56]	–
N.P.	≈ 4	≈ 923–1238^a^	1408–1661 [31]	1.533–1.743 [56]	–

TMM	3.87–4.1	1000–1018	1482–1493	1.48–1.52	1.8–4.4 (20 MHz)

a Values computed using our experimental measurements and the impedance (Z) data reported in Tanoren, et al. [56].

Concerning the speed of sound, as $c_{w}$ is independent of frequency and considering the high water content of the TMM, we assumed $c_{s}$ to be constant with respect to the frequency. This assumption was experimentally validated on agar-based hydrogels between 1 and 10 MHz using different acoustic characterisations techniques [64]. Our experimental values are in the range of previously reported values for phantoms with an agar matrix [20] or gelatin matrix [11] and less than 4% lower than the commonly used value of 1540 m s^−1^ for soft tissues. Of notable interest, the speed of sound of our TMM are within the range of experimental data measured on porcine NP [31] and slightly lower (≈ 5%) than the 1489–1652 m s^−1^ estimated for the AF. If necessary, new components can be used to increase the speed of sound such as glycerol [63] or evaporated milk [19], [20].

Our phantoms show only weak acoustic attenuation at relatively high frequencies (15–35 MHz). To the best of our knowledge, there is no reported value of α for the IVD in the literature and comparison with soft tissues is difficult as characterisations are usually made at lower frequencies as depicted in Table 2. Nonetheless, by extrapolating the power laws $\alpha =a\;f^{n}$, we obtain values that are in agreement with the acoustic attenuation of brain, liver, breasts or prostate but are still significantly lower than the attenuation of tendon or cartilage. At the cost of a trade-off with the reduced optical scattering, more ${\mathrm{TiO}}_{2}$ can be added or new materials incorporated such as silica [20], graphite [62] or glycerol [63] in order to increase the acoustic attenuation. Although the impact of ${\mathrm{TiO}}_{2}$ on the acoustic properties is often not studied or neglected in similar studies [15], [16], we found that while it does not modulate the speed of sound (relative difference = 0.08% ± 0.08%), it significantly increases the acoustic attenuation in our TMM. Some lines of thoughts to explain these differences could lie in the higher frequencies we worked at or the lower intrinsic acoustic attenuation of the agarose matrix with respect to the PVCP matrix of Spirou, et al. [15].

### 4.3 Photoacoustic measurements and limitations

As a first proof of feasibility to use photoacoustic imaging as a new tool to probe IVD and quantify disc degeneration, a phantom composed of 1.25% w/w agarose and 1 mg/mL ${\mathrm{TiO}}_{2}$ was tested on our photoacoustic setup. While representative photoacoustic images were not acquired in this work, the photoacoustic signal profiles presented in Fig. 13 clearly demonstrate the phantoms ability to generate strong, wavelength-dependent photoacoustic responses, which are directly relevant to its imaging performance. The magnitude of the signals obtained between 700 and 900 nm on this TMM and the porcine discs are comparable, thus validating both the ability of our line of TMM to accurately mimic healthy discs and the potential of photoacoustic imaging to probe IVD.

These TMM could be valuable for future research investigating the application of various medical imaging technologies to cartilaginous tissues and IVD. Specifically, they can serve as calibrated ground truths for validating inverse problem solutions in experimental or clinical settings in photoacoustic imaging. However, it is important to emphasise that while our TMM can also mimic the optical properties of cortical and cancellous bones [65] that are anatomically adjacent to the IVD, they are not suitable for replicating the thermo-mechanical properties of these bony structures. This is due to the markedly higher speed of sound and density and significantly lower specific heat capacity of bones compared to our TMM [57].

Only the volumetric coefficient of thermal expansion was not experimentally investigated, resulting in wide estimations of the Grüneisen parameter, which is the main limitation of our study. As summarised in Table 1, $\Gamma_{TMM}$(20 °C) shows values close to $\Gamma_{NP}$ but considerably lower than $\Gamma_{AF}$. The main consequence of this difference would be a poorer signal-to-noise ratio of the acquired PA signal as less optical energy would be converted to pressure but we believe this is not a major issue as a previous study showed that $\Gamma_{TMM}$(T=37 °C) = 1.6 $\;\Gamma_{TMM}$(T=20 °C) for agar hydrogels [66]. Hence, conducting experiments at the physiological temperature of 37 °C, as in *in vivo* conditions, would artificially bring $\Gamma_{TMM}$ closer to its expected values in IVD and significantly enhance the signal-to-noise ratio. These considerations highlight that temperature-dependent corrections could be straightforwardly implemented in future studies to extend the applicability of the phantom to *in vivo* simulations.

Another limitation of our study is the long-term stability of our TMM, which was not evaluated. Qualitatively, we found that storage in airtight containers with distilled water yielded good, albeit unquantified, conservation of the phantoms for up to 2 months.

Finally, in their current form, our phantoms are characterised as homogeneous materials. Depending on the concentrations, they can mimic different IVD compartments as a single layer. Preliminary work has demonstrated that extending this methodology to multilayer phantoms (such as multi-compartments IVD phantom) is feasible and will be the subject of a future study which could be validated through representative photoacoustic images.

## 5 Conclusion

In this paper, we introduced and extensively characterised a new line of tissue-mimicking materials with a ${\mathrm{TiO}}_{2}$-doped agarose matrix to design intervertebral discs photoacoustic phantoms. Empirical constitutive laws were derived for each of the parameters of interest as a function of the components concentration, allowing for the creation of phantoms with tailored properties. This comprehensive characterisation is of particular interest for the creation of relevant phantoms of various soft tissues, extending the applicability of this work beyond intervertebral disc studies to broader photoacoustic imaging applications.

Specifically, phantoms with $C_{agarose}\;\in$ [0.5, 3] %w/w and $C_{{\mathrm{TiO}}_{2}}\;\in$ [0.5, 1.5] mg/mL demonstrate optical properties in the range of the expected values for IVD with thermo-mechanical properties comparable to nuclei pulposi. The relative differences with the annulus fibrosus are small to marginal ($\Delta c_{p}<14\text{\%}$, $\Delta c_{s}<5\text{\%}$ and Δρ<3.5%) and could be mitigated by adding other components if necessary. Comparisons between the experimental photoacoustic peak-to-peak spectra measured on porcine intervertebral discs and one of our phantoms demonstrate the ability of our TMM to accurately mimic healthy IVD. This represents a promising first step towards a future quantitative diagnosis of disc degeneration using photoacoustic imaging but future work needs to further investigate the feasibility of this approach in the operating room.

Due to reduced water content and increased vascularisation, degenerated discs are likely to exhibit higher optical absorption and scattering as well as a slightly increased mass density and acoustic velocity compared to healthy discs. In the scope of disc degeneration quantification using photoacoustic imaging, future works could also focus on developing phantoms with higher agarose concentrations to simulate the early states of degeneration. Concerning the last stages of degeneration, adding a red dye or porcine blood could also be considered to account for the blood present in severely degenerated discs. This flexibility reinforces the phantoms relevance as a baseline model for both healthy and degenerated intervertebral discs studies. Finally, the mechanical and osmotic properties of this line of TMM can be enhanced by adding chondroitine sulfate, collagen or a crosslinking agent to better reproduce the mechanical and biochemical environment of intervertebral tissues.

## CRediT authorship contribution statement

**Roman Allais:** Writing – original draft. **Valentin Espinas:** Resources. **Antoine Capart:** Resources. **Anabela Da Silva:** Supervision. **Olivier Boiron:** Supervision.

## Declaration of competing interest

The authors declare that they have no known competing financial interests or personal relationships that could have appeared to influence the work reported in this paper.

## Data Availability

Data will be made available on request.
